# Endosome maturation during ER stress relies on the ubiquitin-binding domain of histone deacetylase 6

**DOI:** 10.1091/mbc.E25-01-0024

**Published:** 2025-08-13

**Authors:** Katherine M. Piscopo, Brooke Larson, Anna M. Christiansen, Jason M. Perry, Julie Hollien

**Affiliations:** School of Biological Sciences and the Center for Cell and Genome Science, University of Utah, Salt Lake City, UT 84112

## Abstract

Histone deacetylase 6 (HDAC6) helps cells manage misfolded proteins by transporting ubiquitin (UB)-associated structures toward the microtubule organizing center, where they can be sequestered and degraded by lysosomes. Here, we show that when cells are subjected to acute protein-folding stress in the endoplasmic reticulum (ER), HDAC6 depletion results in the appearance of enlarged endosomes that are highly decorated with UB and colocalize with both early and late endosome markers. The C-terminal UB-binding domain and adjacent disordered regions of HDAC6 are necessary and sufficient to rescue this endosomal phenotype in cells lacking endogenous HDAC6. HDAC6 deficiency does not appear to prevent the recruitment of endosomal sorting complexes required for transport (ESCRT), which coordinate endosome maturation. However, overexpression of HDAC6 can reverse endosome phenotypes associated with the depletion of the early ESCRT factor HRS. We speculate that HDAC6 facilitates the packaging and processing of endosomal cargo when the endomembrane system is under stress.

## INTRODUCTION

Histone deacetylase 6 (HDAC6) is a key player in many cellular processes, including cell proliferation and migration, immune responses, and protein homeostasis (Zhu et al., 2023). Mainly cytoplasmic, it deacetylates a variety of highly expressed proteins, including alpha-tubulin (Hubbert et al., 2002; Matsuyama et al., 2002), HSP90 (Kovacs et al., 2005), and cortactin (Zhang et al., 2007). In addition to its catalytic activity, HDAC6 binds ubiquitin (UB) through a C-terminal zinc finger domain (Seigneurin-Berny et al., 2001; Hook et al., 2002), and can also bind the microtubule motor protein dynein (Kawaguchi et al., 2003). These features allow HDAC6 to mediate the trafficking of misfolded, ubiquitinated proteins toward the microtubule-organizing center (MTOC), leading to their sequestration within juxtanuclear aggresomes and their eventual degradation through macroautophagy (Kawaguchi et al., 2003; Iwata et al., 2005). This function of HDAC6 in controlling protein aggregation is thought to underlie its role in neurodegenerative diseases such as Huntingtin’s, Parkinson’s, and Alzheimer’s (Richter-Landsberg and Leyk, 2013; LoPresti, 2020).

Since the discovery of HDAC6’s involvement in aggresome formation, it has become increasingly recognized as an important regulatory link between the UB-proteasome system and macroautophagy (Pandey et al., 2007; Lee et al., 2010) and in the modulation of cytosolic chaperone networks and stress pathways. For example, UB binding by HDAC6 leads to its dissociation from the repressive chaperone complex HSP90-HSF1 (Boyault et al., 2007), and its deacetylase activity can promote the stabilization of HIF-1alpha in the response to hypoxia (Ryu et al., 2017). Although no specific role for HDAC6 in the endoplasmic reticulum (ER) stress response has been described, HDAC6 binds to p97/VCP (Seigneurin-Berny et al., 2001; Boyault et al., 2006), which is involved in the extraction and proteasome-dependent degradation of misfolded ER proteins (Richly et al., 2005), a process termed ER-associated degradation (ERAD) (Sun and Brodsky, 2019). Binding to p97/VCP disrupts HDAC6-UB complexes, leading to a model where the balance between HDAC6 and p97/VCP levels influences the fate of ubiquitinated proteins (Boyault et al., 2006; Li et al., 2017).

Protein-folding stress in the ER can occur through an overwhelming burden of proteins entering the secretory pathway or through dysfunction in the ER protein folding network. The key players in the response to ER stress, known as the unfolded protein response, affect many cellular structures and processes and are involved in a variety of diseases, including neurodegeneration (Almanza et al., 2019; Ghemrawi and Khair, 2020; Celik et al., 2023). One way that cells respond to ER stress is through the IRE1-dependent degradation of the mRNA encoding the biogenesis of lysosome-related organelles complex 1 subunit 1 (BLOC1S1, or BLOS1 [Pu et al., 2015]), whose depletion leads to the juxtanuclear clustering of late endosomes and lysosomes near the MTOC (Hollien et al., 2009; Bae et al., 2019). Overriding the degradation of the *Blos1* mRNA leads to both the dispersion of lysosomes throughout the cell and to the cytosolic accumulation of aggregating proteins, including the mutant Huntingtin protein (Bae et al., 2019; 2022). We have previously proposed that the juxtanuclear clustering of endosomes and lysosomes is beneficial because it increases the ability of these organelles to encapsulate and degrade aggregating proteins, which also traffic to the MTOC via HDAC6. We therefore predicted that depletion of HDAC6 would negate the positive effects of *Blos1* degradation on cell survival during ER stress. Instead, as described here, we found that cells deficient in HDAC6 not only fail to traffic UB foci to the MTOC, but also accumulate endosomes that are stalled in their maturation.

Endosomes resulting from the internalization and fusion of endocytic vesicles from the plasma membrane carry out a complex maturation process before they fuse with lysosomes to complete the degradation of their cargo. Endosome maturation involves trafficking, acidification, formation of intraluminal vesicles, and many coordinated exchanges of membrane-associated proteins. One of the major events is the exchange of the GTPase RAB5, a marker for early endosomes and endocytic vesicles, for RAB7, which is found on late endosomes, lysosomes, and autophagosomes (Borchers et al., 2021). Other key factors that coordinate endosome maturation include the endosomal sorting complexes required for transport (ESCRTs) (Vietri et al., 2020), which sort endocytosed membrane proteins for delivery into endosomes and provide the machinery responsible for formation of intraluminal vesicles. Ubiquitinated cargo proteins on the cytoplasmic surface of the endosomal membrane are typically recognized by the ESCRT-0 complex, which in mammals is composed of HRS and STAM proteins (Bache et al., 2003). Concentrating ESCRT-0 and ubiquitinated proteins in microdomains results in the further recruitment of downstream ESCRT complexes, ultimately leading to the inward invagination and scission of endosomal intraluminal vesicles, and the recycling of ESCRTs and UB (Vietri et al., 2020).

The ER has also been implicated in endosome maturation (Friedman et al., 2013; Wu and Voeltz, 2021), although the exact mechanism for this is unclear. As it does with many organelles, the ER makes close contacts with endosomes (Raiborg et al., 2015) and contributes to their function by exchanging lipids and mediating local endosomal movement and fission (Rocha et al., 2009; Rowland et al., 2014; Jongsma et al., 2016). Here, we describe a new connection between the ER and endosomes, where acute ER stress reveals a dependence on HDAC6 for efficient RAB exchange and endosome maturation.

## RESULTS

### Depletion of HDAC6 leads to the appearance of enlarged, UB-associated endosomes during ER stress

**FIGURE 1:**
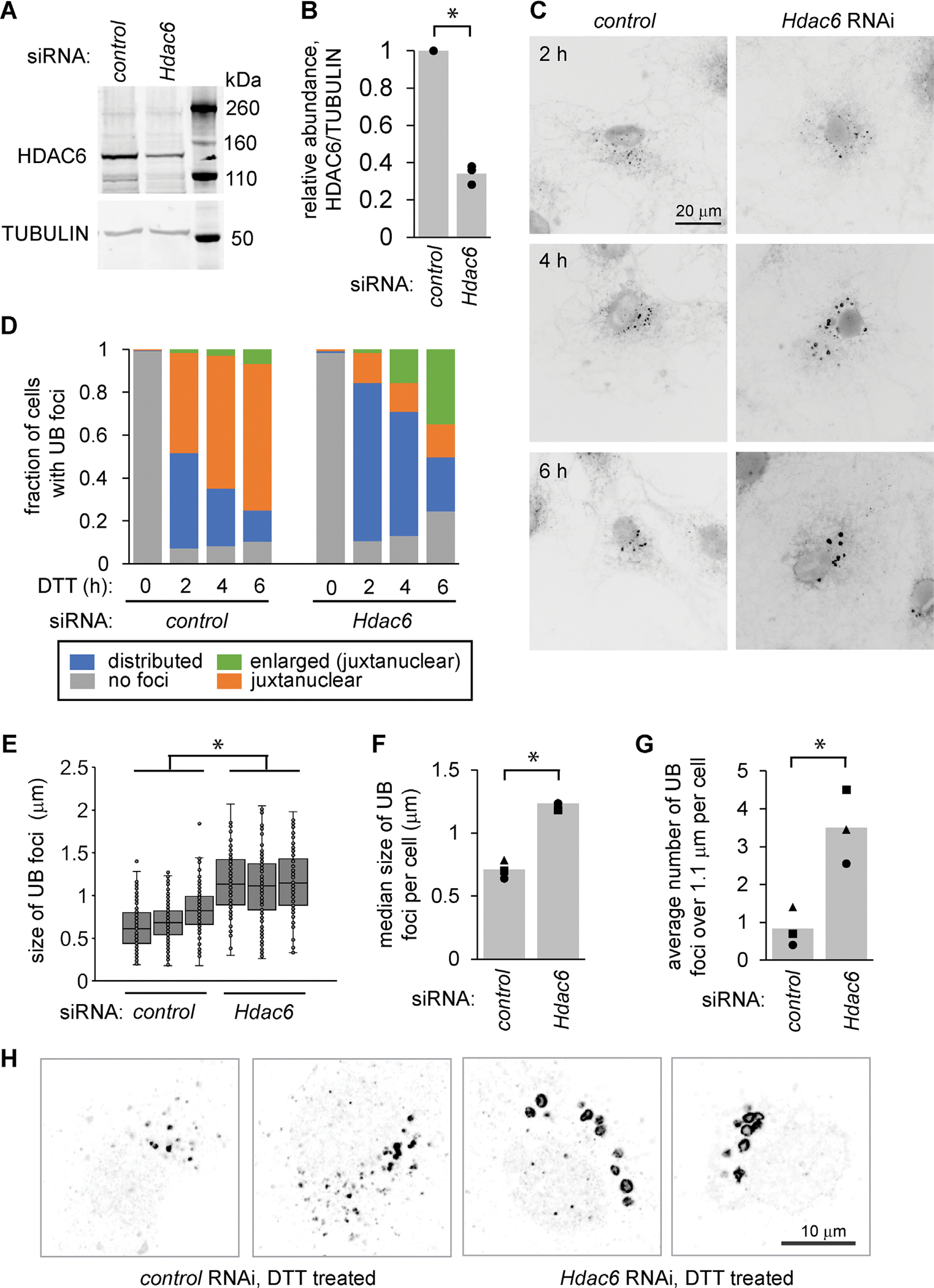
Depletion of HDAC6 leads to enlarged UB structures in MC3T3 cells during ER stress. (A and B) We used siRNA-mediated silencing to deplete HDAC6 from MC3T3 cells for 48 h and measured the knockdown efficiency by immunoblotting. B shows the quantifications for three independent experiments. (C) Following siRNA treatment, we treated cells with 2 mM DTT for 2, 4, and 6 h, and then washed briefly with 60 μg/ml digitonin to release soluble proteins before fixing, staining for UB, and imaging on a wide-field fluorescence microscope. (D) We scored the localization and appearance of UB foci in cells from C (three independent experiments, 60 cells per condition). (E–G) We quantified cells treated as in C following 6 h DTT (three independent experiments, 20 cells per condition). E shows the diameter of all UB foci, F shows the median size of foci per cell in each experiment, and G shows the average number of foci over 1.1 μm per cell. For F and G, markers represent the average of 20 cells for each experiment and bars represent the average of the three independent experiments. (H) Higher resolution confocal images of the juxtanuclear area of cells treated with DTT (2 mM, 6 h) and stained for UB as in C. *p<0.05, paired *t* test.

To explore the role of HDAC6 in the trafficking and management of misfolded proteins during ER stress, we used siRNA-mediated silencing to deplete HDAC6 from MC3T3-E1 cells (Figure 1, A and B), then induced ER stress using DTT, which reduces disulfide bonds. We briefly permeabilized cells with digitonin to release soluble proteins, fixed with paraformaldehyde, permeabilized further with Triton-X, and stained using an antibody for ubiquitinated proteins. As expected, ER stress led to the accumulation of detergent-resistant UB foci, which clustered near the nucleus over time. Depletion of HDAC6 delayed this clustering, resulting in more peripheral UB foci (Figure 1, C and D). After longer periods of stress (up to 6 h), we observed a pronounced increase in the size of the UB foci in the cells depleted of HDAC6 (Figure 1, E–G). We also observed these structures in cells that were not permeabilized before fixing (Supplemental Figure S1A), and in U2OS cells depleted of HDAC6 and treated with thapsigargin (TG), an alternative inducer of ER stress (Supplemental Figure S1, B and C).

**FIGURE 2:**
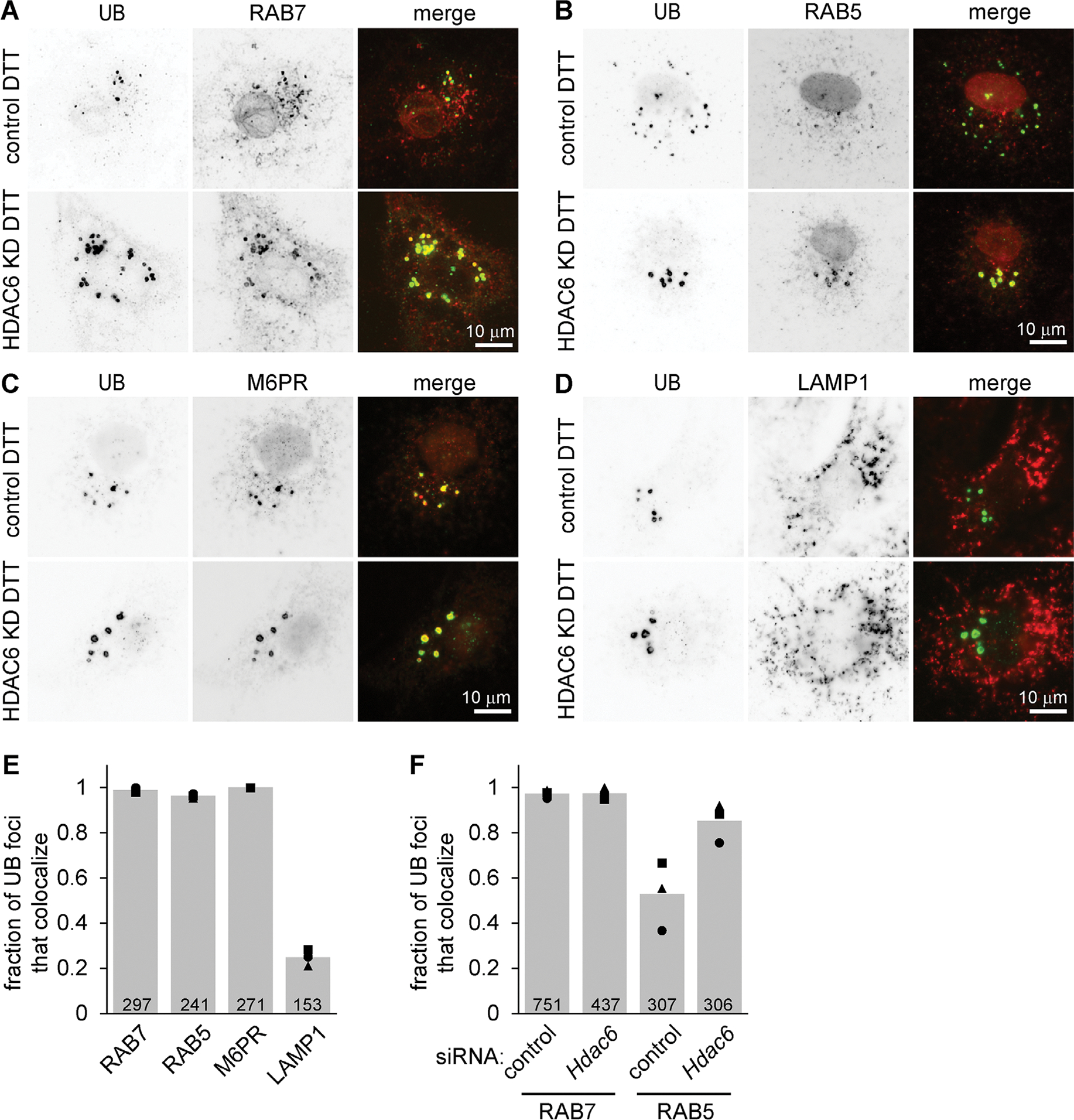
UB structures in cells undergoing ER stress colocalize with endosome markers. (A–D) We depleted MC3T3 cells of HDAC6 using siRNAs, treated with DTT (2 mM, 6 h), washed briefly with 60 μg/ml digitonin, fixed, and stained using antibodies for UB (green) and either RAB7, RAB5, M6PR, or LAMP1 (red). (E) For the cells depleted of HDAC6 and treated with DTT as in A to D, we scored the colocalization of the indicated proteins with each UB structure that had a minimum diameter of 1.1 um. (F) We scored the colocalization of the indicated proteins with each UB structure. For E and F, markers show the average fraction of colocalizing UB foci per cell across 20 cells from one experiment; bars show the average of three independent experiments. The total number of foci quantified is indicated at the bottom of each bar.

These UB structures often displayed a ring-like appearance, consistent with the surface decoration of membrane-bound vesicles or organelles (Figure 1H). To test this idea and identify the structures, we costained cells for UB and a series of markers for organelles in the endomembrane system. The UB foci colocalized with the late endosome markers RAB7 and M6PR, as well as the early endosome marker RAB5, but not with the lysosome marker LAMP1 (Figure 2, A–F). Although ER stress is sometimes associated with an increase in macroautophagy, we did not detect increased LC3B processing following DTT treatment and/or HDAC6 depletion in our experimental conditions (Supplemental Figure S1, D and E), and the UB structures did not colocalize with LC3 (Supplemental Figure S1F), indicating they are unlikely to be autophagosomes. We conclude that these structures are nonstandard endosomes, perhaps stalled in their maturation as discussed below.

### The HDAC6 endosome phenotype is specific for ER stress

**FIGURE 3:**
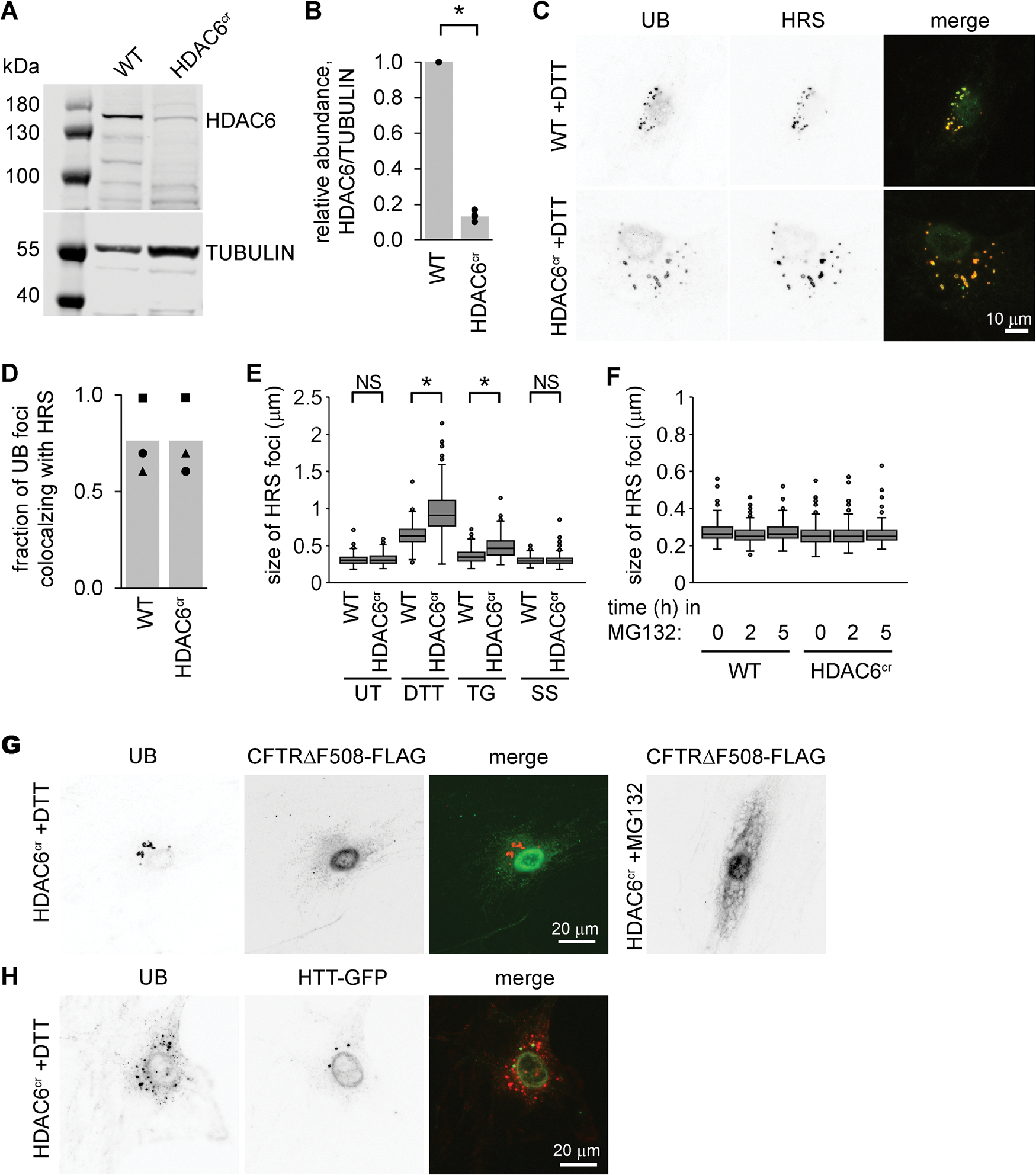
The endosome phenotype of HDAC6-knockout cells appears only during ER stress. (A and B) We used CRISPR/*Cas9* to mutate the HDAC6 locus in MC3T3 cells, and selected a clonal population referred to here as HDAC6^cr^ (see Materials and Methods). We collected protein and measured the levels of HDAC6 by immunoblotting. B shows the quantification of three independent experiments. *p<0.05, paired *t* test; WT, unedited MC3T3 cells. (C) We treated WT and HDAC6^cr^ cells with DTT (2 mM, 5 h), washed briefly with 10 μg/ml digitonin, then fixed and stained using antibodies for UB (green) and HRS (red). (D) We scored the fraction of UB structures that colocalized with HRS foci in cells from C. Markers show the average fraction per cell across 20 cells from one experiment; bars show the average of three independent experiments. (E) We treated WT and HDAC6^cr^ cells with DTT (2 mM, 5 h), TG (2 μM, 18 h), or serum-free media (SS, 18 h). We then stained for HRS as in C and measured the size of the 10 largest HRS foci per cell (three independent experiments, 10 cells per condition for each experiment). *, *p*<0.05, ANOVA with Tukey’s HSD. NS, not significant. (F) We treated WT and HDAC6^cr^ cells with 10 μM MG132 for the indicated times and analyzed HRS foci as in E. (G) We transfected HDAC6^cr^ cells with a plasmid expressing CFTRΔF508-FLAG, treated with DTT (2 mM, 5 h) or MG132 (10 μM, 5 h), washed briefly with 10 μg/ml digitonin, fixed, and stained for UB (red) and FLAG (green). The MG132 image shows typical accumulation of CFTRΔF508-FLAG when its degradation by ERAD is inhibited. (H) We transfected HDAC6^cr^ cells with a plasmid expressing HTT-GFP, treated with DTT (2 mM, 5 h), washed briefly with 10 μg/ml digitonin, fixed, and stained for UB (red) and HTT-GFP (green).

To further explore the role of HDAC6 in endosome biology, we disrupted the endogenous *Hdac6* gene using a CRISPR/CAS9 system, generating a cell line (HDAC6^cr^) with severely reduced HDAC6 levels (Figure 3, A and B). Chemical induction of ER stress using either DTT or TG led to enlarged UB structures in the HDAC6^cr^ cells (Supplemental Figure S2, A and B), similar to those seen in the cells depleted of HDAC6 with siRNAs.

We next asked whether the endosome phenotype of HDAC6-deficient cells occurs only in response to ER stress. We did not observe any differences in RAB7 foci between wild-type and HDAC6^cr^ cells in the absence of stress, but the abundance of foci and high background signals made it difficult to measure subtle size differences reliably (Supplemental Figure S2C). We therefore used antibodies for the early ESCRT factor HRS to examine endosomes in various conditions. As expected for an endosome-associated protein, HRS colocalized with UB in cells treated with DTT (Figure 3, C and D). In the absence of applied stress, or during serum starvation or inhibition of the proteasome with MG132, the HRS foci were similar in size in the wild-type and HDAC6^cr^ cell lines (Figure 3, E and F). When we induced ER stress with either DTT or TG, the HRS foci increased in size in both cell lines but became even more enlarged in the HDAC6^cr^ cells, as seen for the UB foci (Figure 3E).

To test an alternative inducer of ER stress, we transiently overexpressed exon1 of a disease-causing variant of the human Huntingtin protein containing an expanded repeat of 145 glutamine residues and tagged with green fluorescent protein (HTT-GFP). We have previously shown that this protein induces ER stress and its transient expression results in a small number of enlarged RAB7-associated foci per cell ((Bae et al., 2022) and Supplemental Figure S2D). Consistent with a role for HDAC6 in maintaining endosome function during stress, the fraction of cells containing at least one of these enlarged RAB7 structures increased in cells depleted of HDAC6 compared with control cells (Supplemental Figure S2, D and E).

Our data show that HDAC6-deficient cells accumulate UB-associated, enlarged endosomes under conditions that cause ER protein misfolding. One possible explanation for this is that misfolded ER proteins overwhelm the ERAD machinery and are redirected to endosomes and lysosomes for degradation, resulting in the need for additional factors like HDAC6 to accommodate this increased flux. Proteins could reach endosomes via membrane trafficking or through retrotranslocation into the cytosol, followed by uptake into endosomes via microautophagy (Sahu et al., 2011; Chauhan et al., 2019; Wang et al., 2023). Arguing against this idea, however, preventing ubiquitinated protein degradation in the cytosol with MG132 did not affect endosome size in our wild-type MC3T3 or HDAC6^cr^ cells (Figure 3F), and reducing the ability of ERAD substrates to access the cytosol by depleting cells of HRD1 did not appear to alter the DTT-induced endosome phenotype (Supplemental Figure S2, F and G). Furthermore, the enlarged, UB-associated endosomes that accumulated during DTT treatment did not colocalize with transiently expressed CFTRΔF508, a model transmembrane substrate of ERAD (Figure 3G). We did see a small number of UB foci colocalizing with HTT-GFP during ER stress, similar to the RAB7/HTT-GFP foci we observed in the absence of chemical inducers of stress and consistent with HTT-GFP being a substrate for endosomal microautophagy (Bae et al., 2022). However, the vast majority of the DTT-induced, UB-associated endosomes did not colocalize with HTT-GFP (Figure 3H). These data suggest that while strongly correlated with ER stress, the endosome phenotype in HDAC6-deficient cells may not be directly related to the degradation of misfolded proteins in these structures.

### Endosomes from HDAC6-deficient cells subjected to ER stress do not effectively switch from RAB5 to RAB7

**FIGURE 4:**
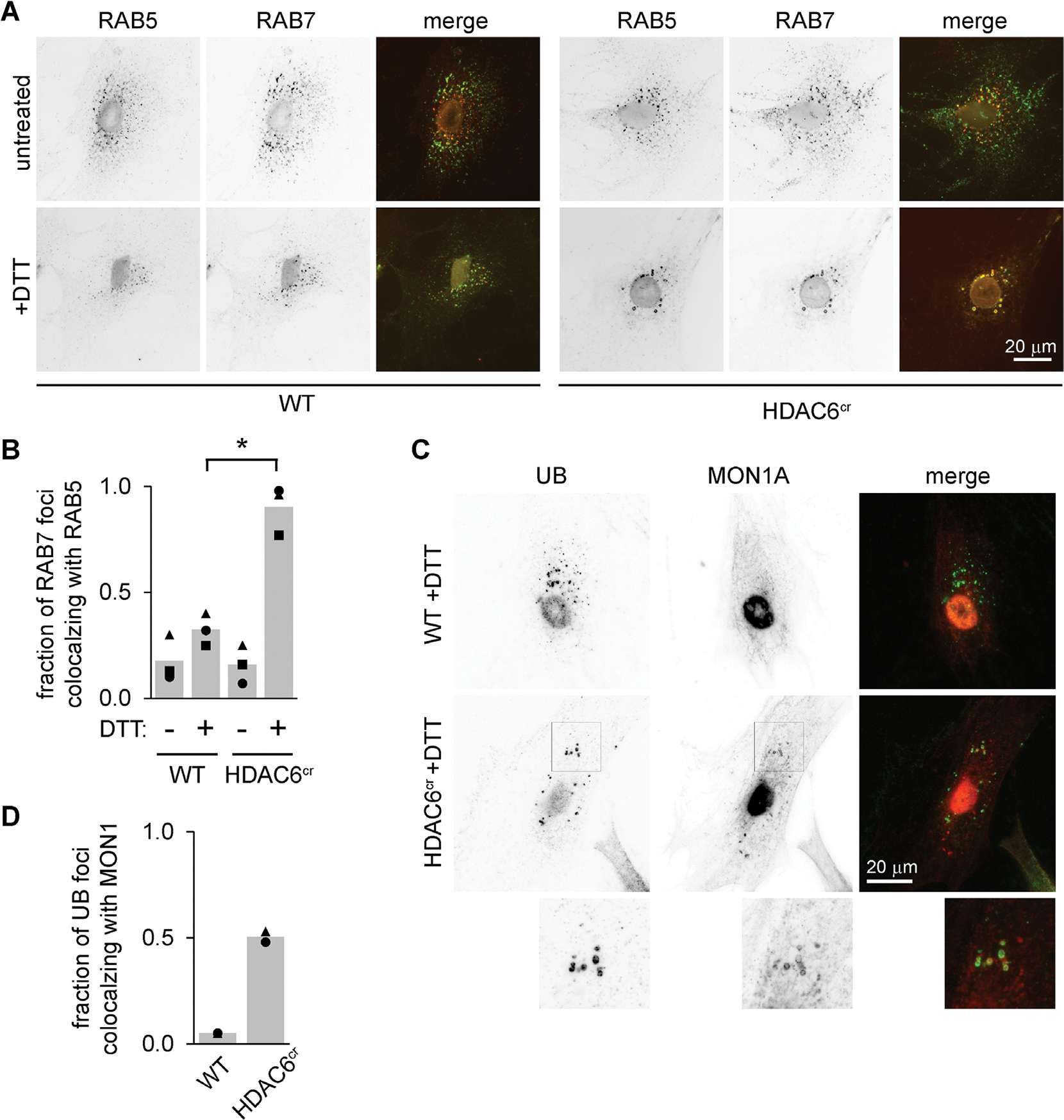
Endosomes in HDAC6-knockout cells fail to exchange RAB5 for RAB7 during ER stress. (A and B) We treated WT and HDAC6^cr^ cells with DTT (2 mM, 5 h), washed briefly with 10 μg/ml digitonin, then fixed and stained using antibodies for RAB5 (green) and RAB7 (red). For B, we scored the 10 brightest RAB7 and RAB5 foci and counted the fraction that colocalized (three independent experiments, 10 cells per condition). *p<0.05, ANOVA with Tukey’s HSD. (C and D) We treated cells as in A, then stained using antibodies for UB (green) and MON1A (red). For D, we scored the 10 brightest UB foci and quantified the fraction that colocalized with MON1A. (two independent experiments, 10 cells per condition).

The presence of both RAB5 and RAB7 on endosomal structures in HDAC6-deficient cells (Figure 2) is unusual, as the maturation of early endosomes into late endosomes/multivesicular bodies involves switching from RAB5 to RAB7 (Rink et al., 2005; Poteryaev et al., 2010). Although UB colocalized very strongly with RAB7 in both the control and HDAC6-depleted cells, RAB5 was less associated with the UB structures in control cells (Figure 2F), suggesting that HDAC6 depletion leads to slower dissociation of RAB5 as endosomes mature. To test this more directly and determine whether this retention of both RAB5 and RAB7 was specific for ER stress, we costained cells with antibodies for RAB5 and RAB7. We found very little overlap between the 10 brightest RAB7 and the 10 brightest RAB5 structures in unstressed cells as expected, and colocalization increased only slightly upon treating control cells with DTT (Figure 4, A and B). However, in HDAC6^cr^ cells treated with DTT, RAB5 and RAB7 strongly colocalized, as predicted by their individual colocalizations with UB (Figures 2 and 4A–B).

A critical regulator of the switch from RAB5 to RAB7 during endosome maturation is the protein complex MON1–CCZ1–RMC1 (Nordmann et al., 2010; van den Boomen et al., 2020; Dehnen et al., 2020), which is recruited to endosomes by RAB5 and acts as a guanine exchange factor that activates RAB7 (Borchers et al., 2021). To test whether MON1A is affected by HDAC6 deficiency, we induced ER stress with DTT and costained cells for UB and MON1A. Although the MON1A signal in wild-type cells was mostly diffuse and not colocalized with UB, MON1A in the HDAC6^cr^ cells substantially colocalized with the enlarged UB foci (Figure 4, C and D). These data suggest that although MON1A is recruited to these endosomes, the RAB switch is delayed, leading to retention of both RABs and MON1 itself on the endosomal membrane.

### The endosomal defect in HDAC6-depleted cells is not a direct consequence of failure to traffic toward the MTOC

**FIGURE 5:**
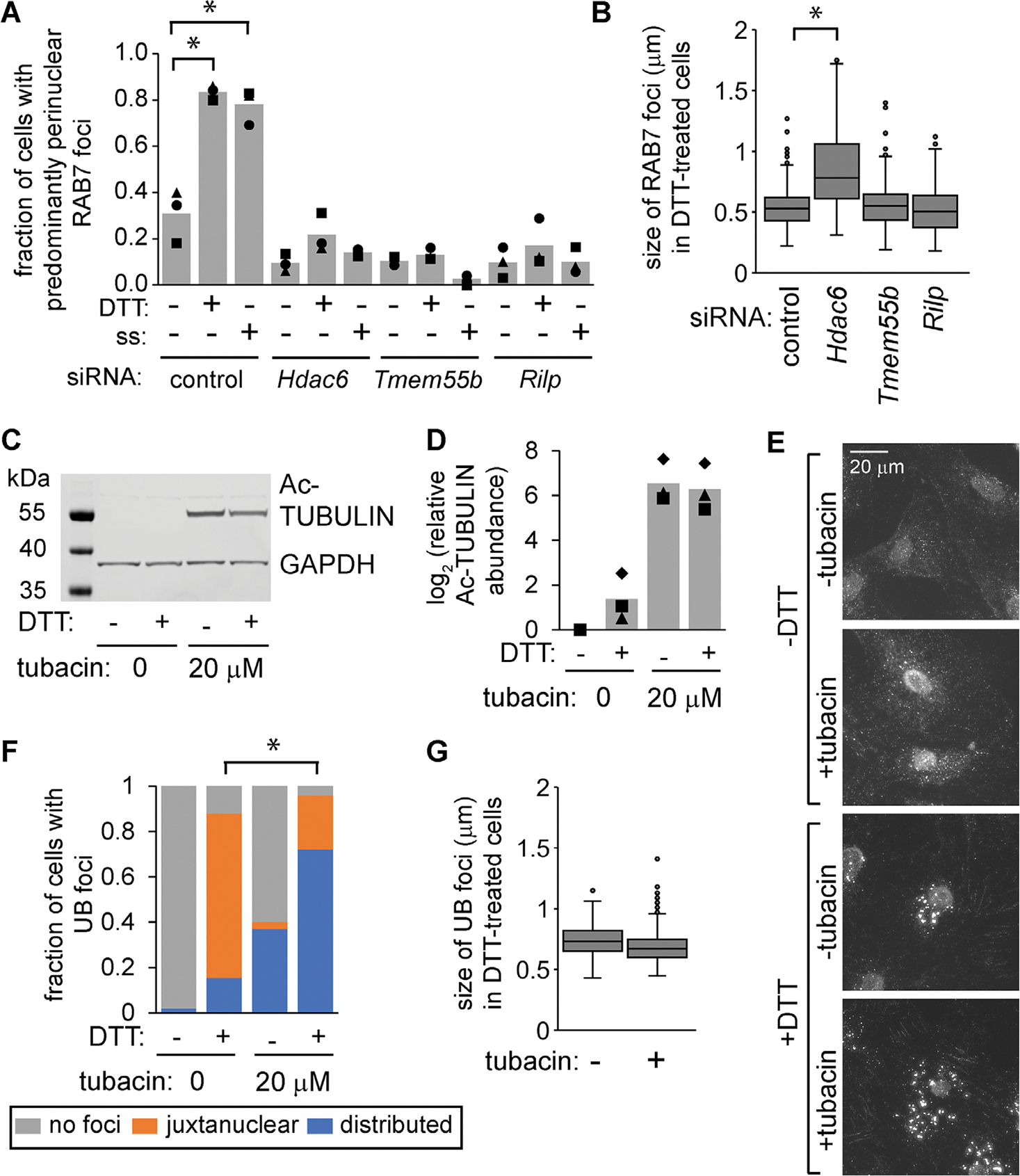
The increase in endosome size in HDAC6-deficient cells is not a direct consequence of failure to traffic to the cell center. (A and B) We used siRNAs to deplete MC3T3 cells of HDAC6, TMEM55b, or RILP for 48 h. We then treated cells with DTT (2 mM, 6 h) or serum-free media (SS, 18 h), fixed and stained using antibodies for RAB7. Representative images are shown in Supplemental Figure S3. For A, we scored the fraction of cells with perinuclear RAB7 foci (three independent experiments, 50 cells per condition for each experiment). For B, we measured the diameter of RAB7 foci (three independent experiments, 30 cells total per condition). *p<0.05, ANOVA with Turkey’s HSD. (C and D) We treated MC3T3 cells with tubacin (20 μM, 6 h) and/or DTT (2 mM, 6 h), collected protein, and measured acetylated TUBULIN by immunoblotting. D shows the quantification of three independent experiments. (E–G) We treated cells as in C, then washed briefly with 60 μg/ml digitonin, fixed, and stained for UB. We then scored the localization of UB foci in F (three independent experiments, 150 cells total per condition), and measured average size of the 10 largest UB foci per cell in G (three independent experiments, 30 cells total per condition).

Endosome maturation is typically accompanied not only by RAB switching but also by microtubule-dependent trafficking toward the cell center (Huotari and Helenius, 2011; Scott et al., 2014). To test whether depletion of HDAC6 affects the maturation of endosomes by preventing their trafficking, we depleted cells of two alternative dynein adaptors that are used to transport late endosome/lysosomes (Jordens et al., 2001; Willett et al., 2017). Although depletion of HDAC6, RILP, or TMEM55b prevented the clustering of RAB7 foci at the MTOC during ER stress (Figure 5A; Supplemental Figure S3), only HDAC6 depletion affected the size of these structures (Figure 5B).

HDAC6 affects microtubule-dependent trafficking in part through its ability to deacetylate alpha-TUBULIN (Hubbert et al., 2002). To further test the connection between trafficking and endosome maturation, we inhibited the deacetylase activity of HDAC6 using tubacin (Haggarty et al., 2003), which led to accumulation of acetylated TUBULIN as expected (Figure 5, C and D). Similarly to HDAC6 depletion, tubacin prevented the juxtanuclear clustering of UB structures during ER stress (Figure 5, E and F). However, tubacin did not affect the size of these UB foci (Figure 5G), supporting the idea that HDAC6 affects endosome size in a manner that is distinct from its role in trafficking.

### An extended C-terminal UB-binding domain of HDAC6 is necessary and sufficient for promoting endosomal maturation during ER stress

**FIGURE 6:**
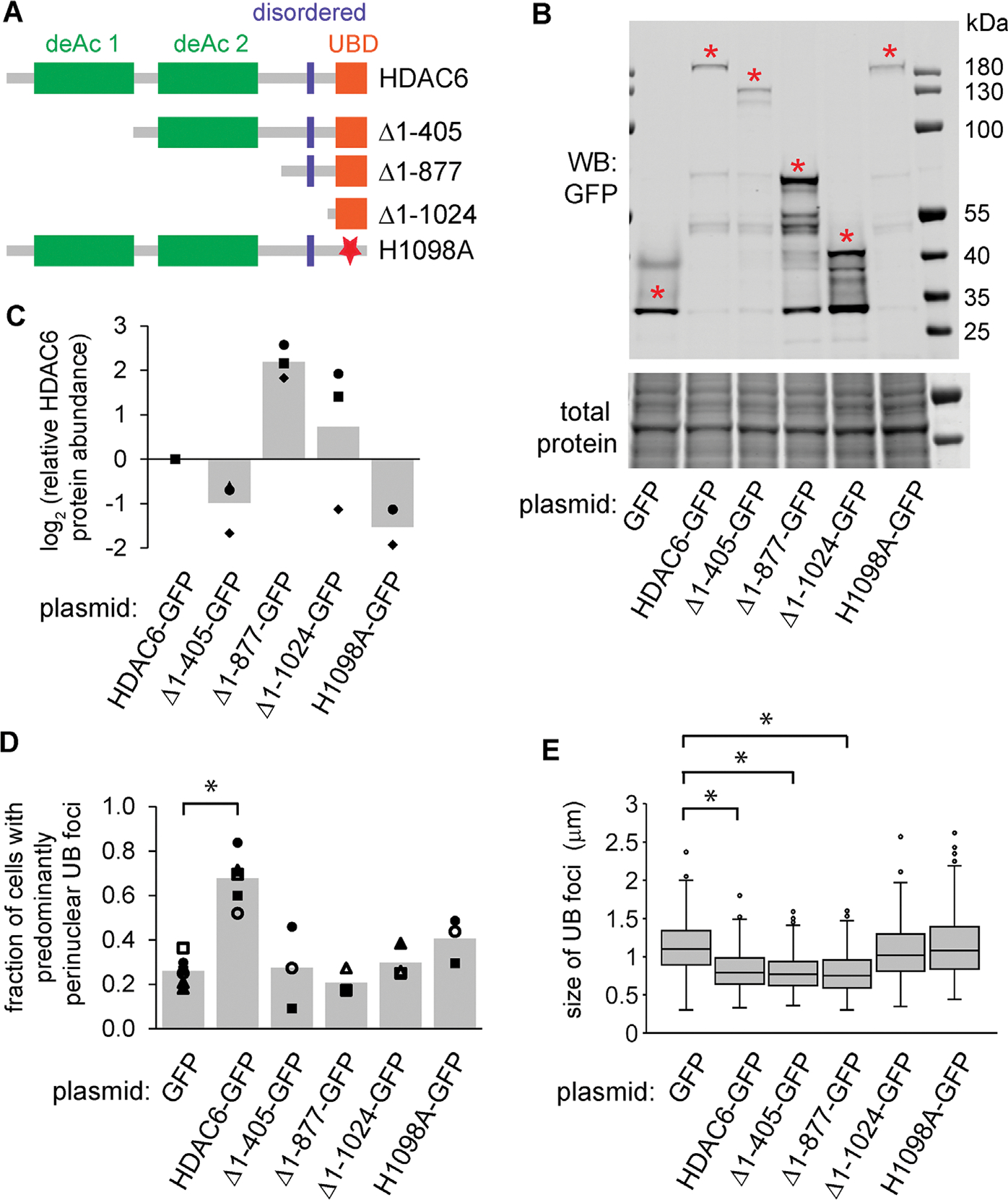
The ubiquitin-binding and adjacent disordered domain of HDAC6 are necessary and sufficient for preventing endosome enlargement in knockout cells. (A) Diagram of HDAC6 domain structure. (B and C) We transfected the HDAC6-GFP variants from A into HDAC6^cr^ cells and measured the protein levels by immunoblotting. Red asterisks indicate the bands of the expected protein sizes, which we quantified in C (three independent replicates). (D and E) We transfected HDAC6^cr^ cells as in B and C, treated with DTT (2 mM, 6 h), washed briefly with 10 μg/ml digitonin, fixed, and stained for UB and GFP. For D, we scored the fraction of cells with perinuclear UB foci (minimum of 68 cells per condition per experiment). For E, we measured the diameter of UB foci (three independent experiments, 10 cells per condition per experiment). *p<0.01, ANOVA with Tukey’s HSD.

To test which domain(s) of HDAC6 are important for preventing endosome enlargement during ER stress, we constructed several truncated and/or mutated variants of HDAC6, each C-terminally tagged with GFP (Figure 6, A–C). As expected, in HDAC6^cr^ cells transfected with GFP and treated with DTT, we observed enlarged and distributed UB foci (Figure 6, D and E). Transfection of full-length HDAC6-GFP fully reversed this phenotype (Figure 6, D and E). Transfection of HDAC6 variants in which the deacetylase domains were deleted, or in which a key UB-binding residue was mutated (Seigneurin-Berny et al., 2001), resulted in dispersed foci (Figure 6D). In contrast, deletion of all but the C-terminal 247 amino acid residues of HDAC6 (1–877), which encompass a disordered domain followed by the UB-binding domain, resulted in an HDAC6 variant that was as effective as the full-length HDAC6 in recovering the normal size distribution of endosomes (Figure 6E). Further truncation, or mutation of the UB-binding domain, resulted in enlarged endosomes as in HDAC6-depleted cells. We conclude that the UBbinding domain and the region just upstream of this domain are necessary and sufficient for efficient endosome maturation during ER stress.

### HDAC6 deficiency does not prevent ESCRT recruitment to endosomes

**FIGURE 7:**
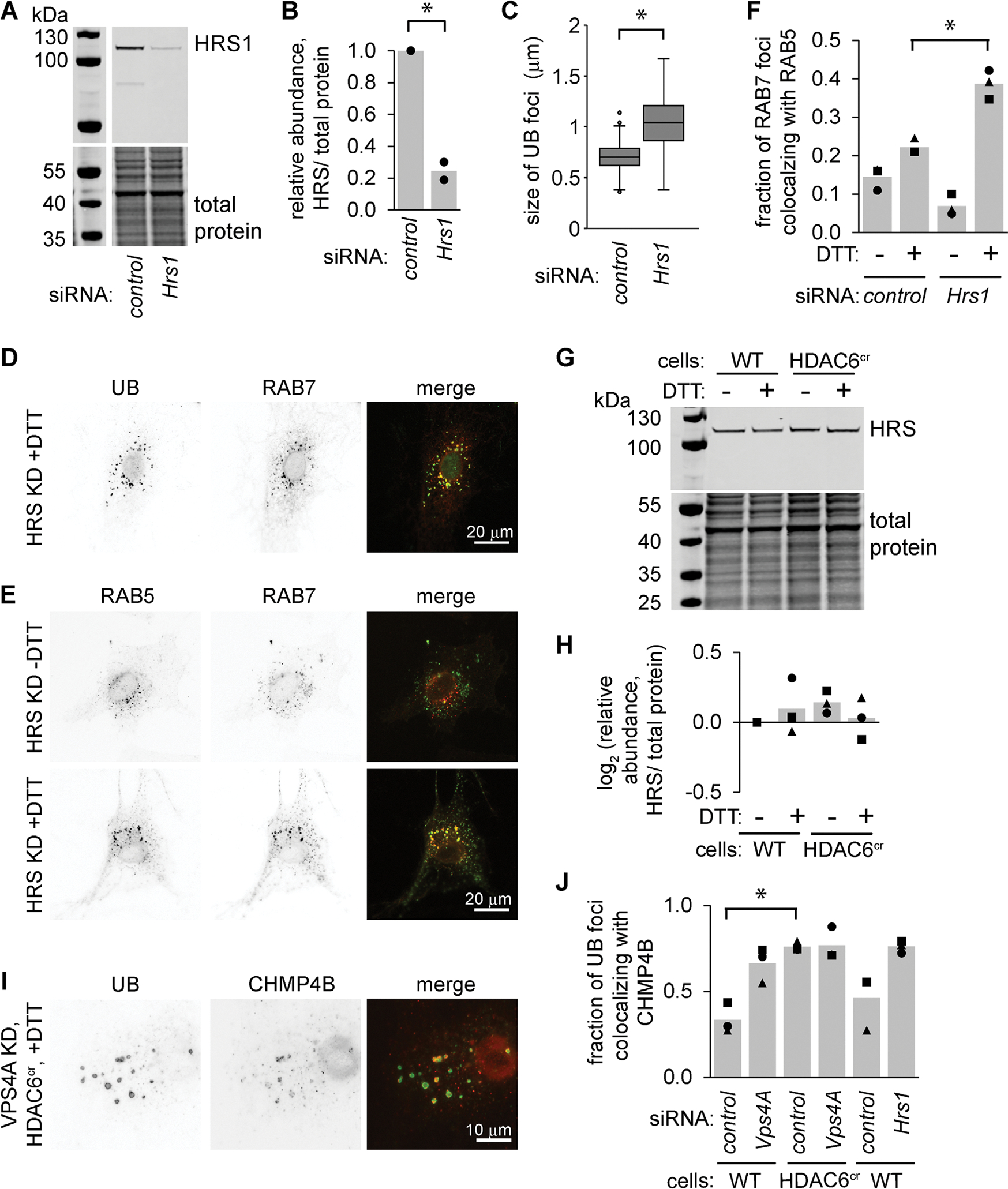
HDAC6- and ESCRT-0–deficient cells display similar phenotypes during stress, but do not prevent ESCRT recruitment to endosomes. (A and B) We used siRNAs to deplete HRS from MC3T3 cells for 48 h and measured the knockdown efficiency by immunoblotting. B shows the quantifications for two independent experiments. *p<0.05, paired *t* test. (C and D) Following siRNA treatment, we treated cells with DTT (2 mM, 4 h), then washed briefly with 60 μg/ml digitonin before fixing and staining for UB. For C, we measured the size of the 10 largest UB foci per DTT-treated cell (three independent experiments,10 cells per condition per experiment). *p<0.05, paired *t* test. D shows representative images with UB (green) and RAB7 (red). (E and F) We treated the cells as in C, washed briefly with 10 μg/ml digitonin, fixed, and stained for RAB5 (green) and RAB7 (red). We scored the 10 brightest RAB7 and RAB5 foci and counted the fraction that co-localized (three independent experiments, 10 cells per condition). *, *p*<0.05, ANOVA with Tukey’s HSD. (G and H) We treated wild-type and HDAC6^cr^ cells with DTT (2 mM, 6 h) and measured HRS abundance by immunoblotting. H shows the quantifications for three independent experiments. (I) We depleted HDAC6^cr^ cells of VPS4A using siRNAs, treated with DTT (2 mM, 5 h), washed briefly with 10 μg/ml digitonin, fixed, and stained for UB (green) and CHMP4B (red). (J) We treated cells as in I, then scored the fraction of UB foci that colocalized with CHIMP4B. Markers show the average fraction of colocalizing UB foci per cell across 20 cells from one experiment; bars show the average of three independent experiments. *p<0.05, paired *t* test with BH correction for multiple pairwise comparisons.

The endosome phenotype described here is reminiscent of cells with defects in the ESCRT machinery, which coordinates cargo packaging and intraluminal vesical formation during endosome maturation. Consistently, we observed enlarged UB structures after depleting cells of the ESCRT-0 factor HRS and subjecting them to ER stress (Figure 7, A–C). These UB foci were colocalized with RAB7 and were similar in appearance to the foci in HDAC6^cr^ cells (Figure 7D). Also, like the HDAC6^cr^ cells, HRS-depleted cells retained both RAB5 and RAB7 on their endosomes during ER stress (Figure 7, E and F).

Despite the similarities in the phenotypes of HRS- versus HDAC6-depleted cells, neither HRS total protein abundance (Figure 7, G and H) nor its association with UB-labeled endosomes (Figure 3, C and D) was affected by HDAC6 deficiency. To assess HDAC6’s potential impact on downstream ESCRT complexes, we costained cells for UB and the ESCRT-III factor CHMP4B. As expected, knockdown of VPS4A, which is important for recycling ESCRT complexes following the formation of intraluminal vesicles, resulted in an increase in the colocalization of detergent-resistant UB foci and CHMP4B. Interestingly, both HDAC6^cr^ cells and cells depleted of HRS also displayed increases in this colocalization (Figure 7, I and J), indicating that ESCRT-III recruitment is not inhibited in HDAC6^cr^ cells.

### HDAC6 enhances endosome maturation when ESCRT-0 function is compromised

**FIGURE 8:**
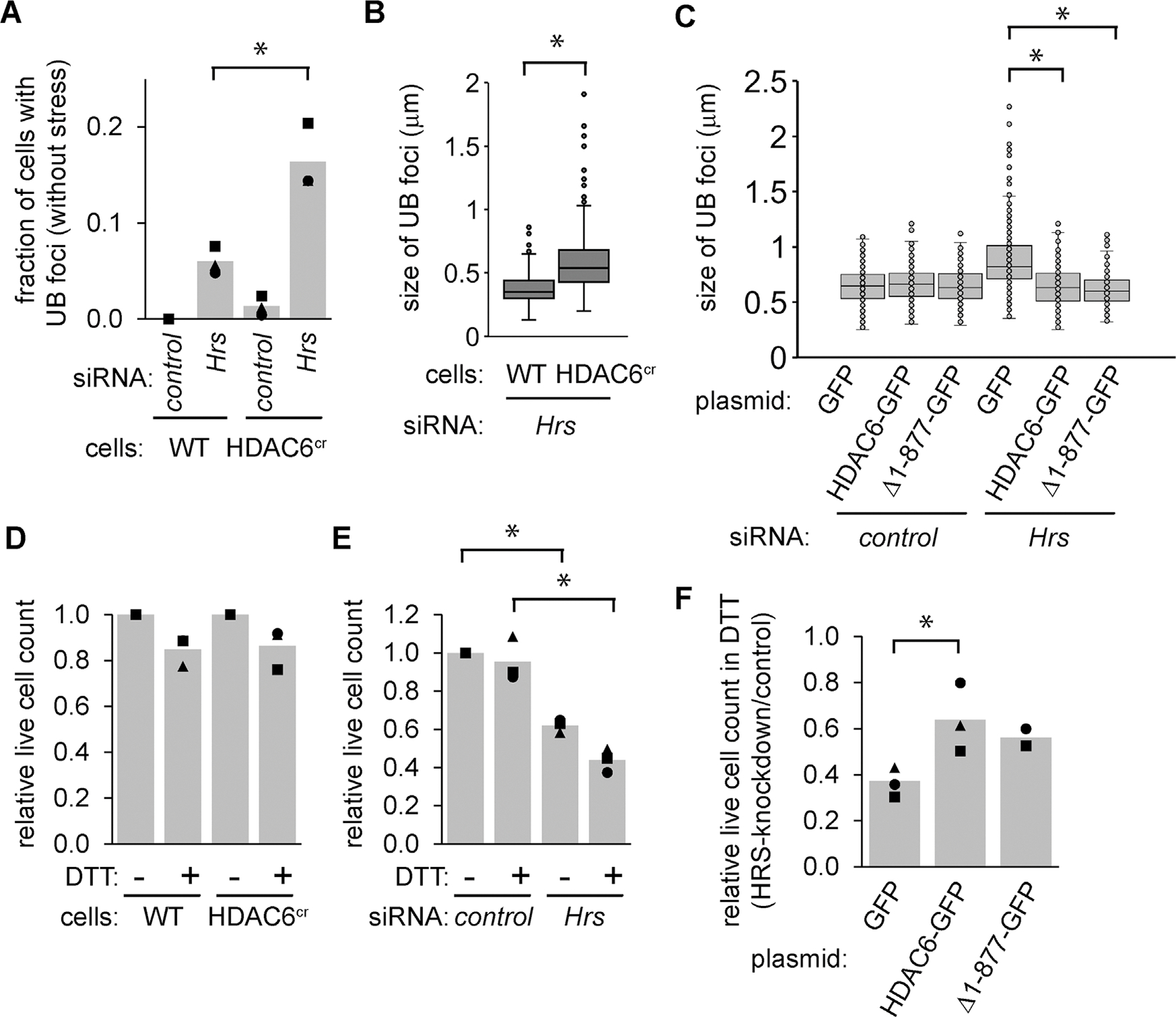
HDAC6 enhances endosome maturation when ESCRT-0 function is compromised. (A) We used siRNAs to deplete HRS from WT and HDAC6^cr^ cells for 48 h, then washed briefly with 60 μg/ml digitonin before fixing and staining for UB. We counted the fraction of cells containing UB foci (three independent experiments, 250 cells per condition). *p<0.05, paired *t* test. (B) We measured the diameter of the 10 largest UB foci from cells in A (three independent experiments,10 cells per condition per experiment). *p<0.05, *t* test. (C) We transfected MC3T3 cells with the indicated HDAC6-GFP variants from Figure 5, allowed cells to recover for 48 h, then used siRNAs to deplete HRS for 48 h. We then treated with DTT (2 mM, 4 h), washed with 10 μg/ml digitonin, fixed and stained for UB and GFP. We measured the diameter of the 10 largest UB foci per cell for cells that were positive for GFP immunostaining (three independent experiments, 10 cells per condition per experiment). *p<0.05, ANOVA with Tukey’s HSD. (D) We treated WT and HDAC6^cr^ cells with DTT (2 mM, 5 h), then fixed and mounted on slides with DAPI. We counted the relative number of intact nuclei in each condition (three independent experiments, minimum of 200 cells per condition per experiment). (E) We used siRNAs to deplete MC3T3 cells of HRS, then quantified cells as in D. (F) We used slides from C to evaluate relative viability of cells, as in D. For E and F: *p<0.05, ANOVA with Tukey’s HSD. Similar comparisons for D were not significant.

To better understand the relationship between HDAC6 and the ESCRTs, we tested the effects of codepletion. Although HDAC6-deficienct cells displayed no obvious phenotypes in the absence of applied stress, a small fraction of unstressed HRS-depleted cells (~5–10%) contained UB foci (Figure 8A). When we depleted HRS from the HDAC6^cr^ cells, more cells contained these foci, and the foci were larger compared with those seen with HRS depletion alone (Figure 8, A and B). This suggested that although HDAC6 is not critical for endosome maturation in the absence of stress, it does become important when ESCRT function is compromised.

To further test this idea, we transfected cells with either full-length HDAC6-GFP or the truncated version that includes the UBbinding domain and disordered region as described in Figure 6. We then depleted cells of HRS, induced ER stress with DTT, and stained for GFP and UB. In cells transfected with GFP, HRS depletion led to enlarged UB structures, as expected. However, in cells that were transfected with the full-length or truncated HDAC6, the size of these structures was not affected by HRS depletion (Figure 8C).

Remarkably, we also observed this mitigating effect of HDAC6 overexpression in cell survival during ER stress. Unlike HDAC6 deficiency, HRS depletion led to a loss of proliferation and viability that was exacerbated by ER stress (Figure 8, D and E), as reported previously (Bae et al., 2019). However, when we transfected cells with HDAC6-GFP before depleting HRS, more cells survived the DTT treatment (Figure 8F).

## DISCUSSION

Here, we describe a new function for HDAC6 in the maintenance and maturation of endosomes during ER stress. Depletion of HDAC6 results in large, immature endosomes, which are decorated with UB and associated with both early and late markers of endosomes, but not with the lysosome marker LAMP1. The dramatically increased size of these endosomes may be explained by their retention of RAB5, which would allow for new cargo to continually arrive from the Golgi and/or plasma membrane (Wegner et al., 2010; Russell et al., 2012), coupled with their failure to complete the switch to RAB7, reach a fully mature state, and fuse with lysosomes. Surprisingly, although endosome trafficking on microtubules is associated with their maturation (Huotari and Helenius, 2011; Scott et al., 2014), the endosomal phenotype described here appears to be unrelated to HDAC6’s known functions in dynein-dependent trafficking and TUBULIN deacetylation. Furthermore, although HDAC6 has been implicated in the maturation of autophagosomes by assembling an F-ACTIN network and promoting lysosomal fusion, this effect is dependent on its catalytic activity (Lee et al., 2010), which is dispensable for rescuing the endosomal phenotype described here. Instead, only the UB-binding domain and a neighboring disordered domain of HDAC6 are required to promote the efficient maturation of endosomes during ER stress.

The similarities between HDAC6 and the early ESCRT machinery may provide clues to the mechanism by which HDAC6 promotes endosome maturation. Cells depleted of either HDAC6 or the ESCRT-0 factor HRS show very similar endosomal phenotypes during ER stress, and when codepleted the number and size of UB-associated endosomes increase even in the absence of applied stressors. Furthermore, HDAC6 overexpression can counteract the detrimental effects of HRS depletion, including the endosome defects and the substantial cell death that occurs in ESCRT-deficient cells subjected to ER stress. ESCRT-0 was recently shown to form condensates in association with UB, facilitating the clustering and packaging of cargo proteins at the limiting membrane of the endosome (Banjade et al., 2022). The requirement for both UB-binding and the disordered domain of HDAC6 in the ER stress endosome phenotype suggests that HDAC6 may similarly participate in this process by promoting UB cargo clustering and condensation. Conversely, if endosomes are overloaded with UB during stress, HDAC6 may provide a shielding function, restricting ESCRT clustering to certain microdomains and facilitating their efficient assembly. Somewhat complicating these potential mechanisms, however, is the fact that we have not been able to detect HDAC6 colocalizing with endosomes, either by fluorescence microscopy of live cells expressing HDAC6-GFP or by immunofluorescence. It may be that its interaction with these structures is transient or weak, or that only a small fraction of HDAC6 molecules localize to endosomes. Alternatively, HDAC6 may promote endosome maturation more indirectly, perhaps by regulating the availability and type of ubiquitinated proteins that are targeted to endosomes.

Despite the fact that this endosome phenotype arises in response to acute ER protein misfolding, and that endosomes are known to uptake misfolded proteins for subsequent lysosomal degradation, we do not find evidence for the excessive endosomal accumulation of misfolded proteins in our HDAC6-knockout cells during stress. The enlarged endosomes do not strongly colocalize with the classic ERAD substrate CFTRΔF508, nor with the aggregation-prone Huntingtin protein, which can be detected in endosomes in the absence of applied stress. Furthermore, HDAC6 deficiency does not affect the viability of cells treated with strong chemical inducers of ER stress, suggesting that the defect in endosome maturation described here does not directly exacerbate the protein misfolding problems in the ER. We therefore speculate that when the ER is subjected to stress, its constitutive role in promoting endosome maturation (Wu and Voeltz, 2021) is compromised, leading to the reliance on additional factors, including HDAC6. In addition, as proteins misfold in the ER, the increased flux through ERAD pathways may redirect p97/VCP from its role in dissociating HDAC6–UB complexes (Boyault et al., 2006), resulting in further activation of this HDAC6 function. Understanding exactly how this works awaits further investigation into the mutual regulation of these organelles.

## MATERIALS AND METHODS

### Request a protocol through *Bio-protocol*

#### Cell culture and RNAi

We cultured MC3T3-E1 cells (ATCC) in MEMα media containing nucleosides, L-glutamine, and no ascorbic acid (Life Technologies) with 10% FBS at 37 C and 5% CO_2_. We cultured U2OS cells in DMEM containing high glucose, L-glutamine, and sodium pyruvate (Genesee Scientific) with 10% FBS at 37°C and 5% CO_2_.

For all siRNA-mediated silencing, we transfected the cells with a mix of two siRNAs (Sigma-Aldrich) per target mRNA using the transfection reagent RNAi MAXX (Invitrogen). For HDAC6 silencing in MC3T3 cells, one of the two siRNAs was more effective than the mixture and was used for subsequent knockdowns (Figure 2 and beyond). We used commercial nontargeting siRNAs (Qiagen or Sigma-Aldrich) as controls. We waited for 48 h to allow for degradation of the target mRNA and protein before assaying.

For transient DNA transfections, we transfected 0.5 μg (for HTT-GFP) or 2 μg (for all other plasmids) using Lipofectamine 2000 (Invitrogen). We incubated the cells with the transfection mixes at 37°C for 2 h, then changed the media and allowed cells to recover for at least 48 h before assaying.

#### Immunostaining and microscopy

For immunostaining, we grew cells on glass coverslips. All fixation, permeabilization, and staining solutions were made in PBS containing 1 mM MgCl. All antibody solutions were made in 2% BSA, 0.02% tween-20, and 1 mM MgCl in PBS. Where noted, we prepermeabilized cells before fixation with either 10 or 60 μg/ml digitonin (4°C, 15 min). We then fixed the cells with 4% paraformaldehyde (37°C, 15 min) and permeabilized with 0.2% Triton-X (room temperature [RT], 20 min). We then incubated the coverslips with 2% BSA and 0.02% tween-20 (RT, 10 min) and stained them with primary antibodies. Most antibodies were incubated for 1 h at RT, except for RAB7, RAB5, and M6PR, which were incubated overnight at RT. We washed with 0.02% tween-20 (RT, three 10-min washes, 30 min total), incubated with secondary antibodies, washed again, and mounted coverslips on slides using ProLong Diamond Antifade mountant with DAPI (Invitrogen).

We imaged the cells at RT using either an Olympus IX-51 inverted microscope with a 60X (NA 1.25) oil objective, an Olympus DP23 Monochrome camera, and the CellSens Standard v3 acquisition software, or an Olympus IX-81 inverted microscope with 60X (NA 1.42) oil objective, an ORCA-FLASH 4.0LT+ SCMOS camera, and the CellSens dimension 4.1.1 acquisition software. For confocal microscopy we used a Zeiss LSM 880 Airyscan with a PlanApochromat 63X (NA 1.4) DIC M27 oil objective, a Zeiss LSM T-PMT imager, and ZEN 2.3 SP1 FP3 (black) acquisition software. For image analysis, a researcher blinded to the identity of the samples quantified the cells using the CellSens software. UB and RAB-labeled endosomes were typically circular, and we measured their diameters. Colocalization was scored for structures over a predefined intensity threshold or for the 10 brightest structures in the cell (for RAB5/7). For endosome or UB localization, blinded researchers scored cells where over 50% of foci were clustered on one side of the nucleus as “predominantly perinuclear.”

#### Immunoblotting

To harvest proteins, we trypsinized and collected cells, and lysed in RIPA buffer (25 mM Tris, pH 7.6, 150 mM NaCl, 1% NP-40, 1% Na-deoxycholate, and 0.1% SDS) with protease inhibitors (Thermo Fisher Scientific, A32953). We used the Pierce BCA protein assay kit (Thermo Fisher Scientific, 23227) to measure protein concentrations, using BSA (Thermo Fisher Scientific, 23210) to determine a standard curve. We then resolved proteins using either 4 to 12% (for GFP, HDAC6, Ac-TUBULIN, and HRS) or 12% (for LC3B) polyacrylamide NuPage Bis–Tris gels. We transferred proteins to nitrocellulose membranes and incubated for 1 h in either Intercept TBS blocking buffer (LI-COR 927–60001, used for HDAC6, LC3B, Ac-TUBULIN, and HRS) or 5% dry milk diluted in TBST (20 mM Tris Base, 150 mM NaCl, 0.02% Tween20, pH 7.4, for GFP) at RT. We then incubated with primary antibodies (see Table 1) at 4°C overnight, washed, incubated with secondary antibodies (1 h, RT), and scanned with a LiCor Odyssey CLx Imager. We quantified band intensities using the LiCor Image Studio software.

#### HDAC6 CRISPR/CAS9-knockout cells

We identified potential HDAC6 guide RNAs using the Genescript database and subcloned these into pSpCas9(BB)-2A-GFP (PX458), a gift from Feng Zhang (Addgene plasmid, #48138). We confirmed the resulting plasmids by sequencing (University of Utah Health Science Core Facilities DNA sequencing Labs).

We performed two rounds of CRISPR editing, each time separately transfecting three HDAC6 guide plasmids into MC3T3-E1 cells. After 48 h, we used a Sony FACS MA900 cell sorter to distribute single GFP-expressing cells into wells in 96-well plates. We grew these monoclonal cell populations and screened them by subjecting them to ER stress (2 mM DTT, 5 h), immunostaining for UB, and assessing the HDAC6-deficient phenotype of enlarged UB foci. For clones that displayed this phenotype, we isolated genomic DNA using Quick-DNA Miniprep Plus kits (Zymo Research D4068), amplified the sequence surrounding the expected CAS9 cut sites by PCR, and sequenced the products. The guide RNA that resulted in the most successful editing (GGGCCGCTGTGTGTCCTTTC) targeted exon five of HDAC6. After two rounds of editing, this guide RNA resulted in extensive mutation in the area of the expected cleavage site with multiple peaks suggesting differential editing of genomic copies. One of these clones was selected as the HDAC6^cr^ cell line used here.

#### Plasmids

We obtained the HTT(Q145)-GFP plasmid from the HD Community biorepository (ref #CHDI-90000040) and transferred the coding sequence into an expression vector as described previously (Bae et al., 2022). We received a human CFTR expression plasmid as a gift from Sonya Neal (Origene RC216476) and introduced the mutation removing the codon for F508 using fusion PCR, followed by confirmation by sequencing.

For expression of HDAC6, we used PCR to amplify the full-length open reading frame of mouse HDAC6 from a cDNA template, subcloned into a vector containing the EF1alpha promoter and C-terminal GFP tag, and confirmed by sequencing. For HDAC6 domain mutants, we used the full-length expression plasmid as a template, designed primers flanking the specified amino acid positions, and subcloned the resulting fragments into the same vector backbone as the full-length HDAC6 plasmid.

#### Quantitative PCR

To check the knockdown of HRD1 in Supplemental Figure S2, we carried out RNAi as described above, then collected and purified total RNA using Quick RNA miniprep kits (Zymo Research). We synthesized cDNA using Moloney murine leukemia virus reverse transcriptase (New England Biolabs) and T18 as a primer. We then measured relative RNA abundance for *Hrd1* and the housekeeping control *Rpl19* using a QuantStudio 3 real-time quantitative PCR machine (Life Technologies), with SYBR green as the fluorescent dye. We measured each sample in triplicate and quantified relative to a serially diluted standard curve.

## Supplementary Material

supplement

## Figures and Tables

**TABLE 1.** Antibodies used in this study.

Primary antibodies	Source	Reference number	Application	Working concentration

FLAG	Sigma	F7425	ICC	1:200
GFP	Aves Labs	GFP-1020	ICC	1:1000
Ubiquitinated proteins (FK1)	Genesee	04-262	ICC	1:250
RAB7	Cell Signaling Technology	D95F2	ICC	1:100
M6PR	Cell Signaling Technology	14364	ICC	1:25
LAMP1	DSHB	1D4B	ICC	1:30
LC3B	Proteintech	14600-1-ap	ICC and IB	1:250 (ICC) or 1:1000 (IB)
HRS	Proteintech	10390-1-AP	ICC and IB	1:100 (ICC) or 1:5000 (IB)
MON1A	Cusabio	CSB-PA801242LA01HU	ICC	1:50
CHMP4B	Proteintech	13683-1-AP	ICC	1:300
RAB5	Cell Signaling Technology	2143	ICC	1:25
*α*-Tubulin	Cell Signaling Technology	2144S	IB	1:5000
GFP	Invitrogen Molecular probes	A-6455	IB	1:7500
HDAC6	Novus	NB100-56343	IB	1:200
GAPDH	ProSci	3783	IB	1:1000

Secondary antibodies	Source	Reference number	Application	Working concentration

Alexa-Fluor 488 anti-mouse IgG	Invitrogen	A21202	ICC	1:1000
Alexa-Fluor 488 anti-mouse IgM	Invitrogen	A21042	ICC	1:1000
Alexa-Fluor 488 anti-chicken IgY	Jackson ImmunoResearch	103-545-155	ICC	1:1500
Alexa-Fluor 488 anti-rabbit IgG	Invitrogen	A11008	ICC	1:1000
Alexa-Fluor 532 anti-rabbit IgG	Invitrogen	A11009	ICC	1:1000
Alexa-Fluor 555 anti-rat IgG	Invitrogen	A21434	ICC	1:1000
DyLight 550 anti-mouse IgM	Thermo Fisher Scientific	SA5-10151	ICC	1:1000
IRDye 800CW Goat anti-Rabbit IgG	Licor	926-32211	IB	1:10,000

## SIGNIFICANCE STATEMENT

- HDAC6 is a cytoplasmic deacetylase and UB-binding protein responsible for the microtubule-dependent trafficking of protein aggregates.
- Here, the authors show that during stress in the ER, HDAC6 also promotes the maturation of endosomes in a manner that does not depend on its catalytic activity or its function in trafficking.
- This unexpected role for HDAC6 may allow cells to preserve the function of endosomes when the ER is compromised.

## Abbreviations used:

BLOS1: biogenesis of lysosome-related organelles complex 1 subunit 1
BSA: bovine serum albumin
CFTR: Cystic fibrosis transmembrane conductance regulator
CHMP: Charged multivesicular body protein
DTT: dithiothreitol
ER: endoplasmic reticulum
ERAD: ER-associated degradation
ESCRT: endosomal sorting complexes required for transport
HDAC6: histone deacetylase 6
HRS: hepatocyte growth factor-regulated tyrosine kinase substrate
LAMP1: lysosome-associated membrane glycoprotein 1
LC3: microtubule-associated protein 1A/1B-light chain 3
M6PR: mannose 6-phosphate receptor
MTOC: microtubule-organizing center
RILP: Rab-interacting lysosomal protein
TG: thapsigargin
UB: ubiquitin
VPS4A: Vacuolar protein sorting-associated protein 4A
