# Supplementary material for: Endosome maturation during ER stress relies on the ubiquitin-binding domain of histone deacetylase 6
Source: Mol Biol Cell. Author manuscript; Available in PMC 2026 Jan 30. (PMC12444907; doi:10.1091/mbc.E25-01-0024)
Supplement: supplement [file NIHMS2133975-supplement-supplement.pdf]

Supplemental Materials

Molecular Biology of the Cell

Piscopo *et al*.

Supplementary Figure Legends for "Endosome maturation during ER stress relies on the ubiquitin binding domain of Histone Deacetylase 6" by Katherine M. Piscopo, Brooke Larson, Anna M. Christiansen, Jason M. Perry, and Julie Hollien

Figure S1. HDAC6 depletion leads to enlarged UB foci during ER stress in U2OS cells and does not induce macroautohaghy. (A) We prepared control and HDAC6^{cr} knockout cells (see later figures), induced ER stress with DTT (2 mM, 6 h), fixed cells without pre-treating with digitonin, and stained for UB. (B) We used siRNAs to deplete HDAC6 from U2OS cells for 48 h, treated with thapsigargin (TG, 2 µM, 18 h), permeabilized with 60 µg/ml digitonin, fixed, and stained for UB. (C) We measured the diameter of UB foci in cells from B (3 independent experiments, 30 cells per experiment). * $p<0.05$, paired t test. (D,E) We depleted MC3T3 cells of HDAC6 and treated them with either DTT (2 mM, 6 h) or chloroquine (CQ, 60 µM, 4 h). We measured LC3B processing by immunoblotting. E shows the quantification of three independent experiments. *, $p<0.05$, ANOVA with Tukey's HSD. (F) We treated cells as in D-E, washed them with 60 µg/ml digitonin, fixed, and stained using antibodies for UB (green) and LC3 (red). The CQ treatment is used here as control for the antibody, which otherwise showed only very faint signal due to the lack of significant macroautophagy induction.

Figure S2. HDAC6^{cr} cells show delayed endosome maturation during ER stress. (A, B) We treated WT and HDAC6^{cr} cells with DTT (2 mM, 5 h) or thapsigargin (TG, 2 ᴍM, 18 h) washed briefly with 60 µg/mL digitonin, then fixed and stained for UB. We then measured the diameter of the 10 largest UB foci per cell (3 independent experiments, 10 cells per condition per experiment). (C) We briefly washed untreated wild-type and HDAC6^{cr} cells with 60 µg/mL digitonin, then fixed and stained using an antibody for RAB7. (D,E) Using siRNAs, we depleted MC3T3 cells of *Hdac6* for 48 hours, then transfected the cells with a plasmid expressing HTT-GFP. After allowing cells to recover for 48 hr, we washed with 10 µg/ml digitonin, fixed, and stained RAB7 and GFP. We then counted the number of cells with at least one enlarged RAB7 structure and scored according to whether they also expressed GFP (2 independent experiments, 50 cells per condition). (F) We used siRNA-mediated silencing to deplete HRD1 from MC3T3 cells for 48 h and measured the knockdown efficiency by qPCR. (G) We depleted WT and HDAC6cr cells of Hrd1 as in F, treated with DTT (2 mM, 5 h), fixed and stained for UB, and counted the number of cells that had enlarged UB foci (3 independent experiments, 100 cells per condition).

Figure S3. Representative images of knockdown cells in various conditions. Representative images of RAB7 foci, for experiments described in Fig. 5A and B.

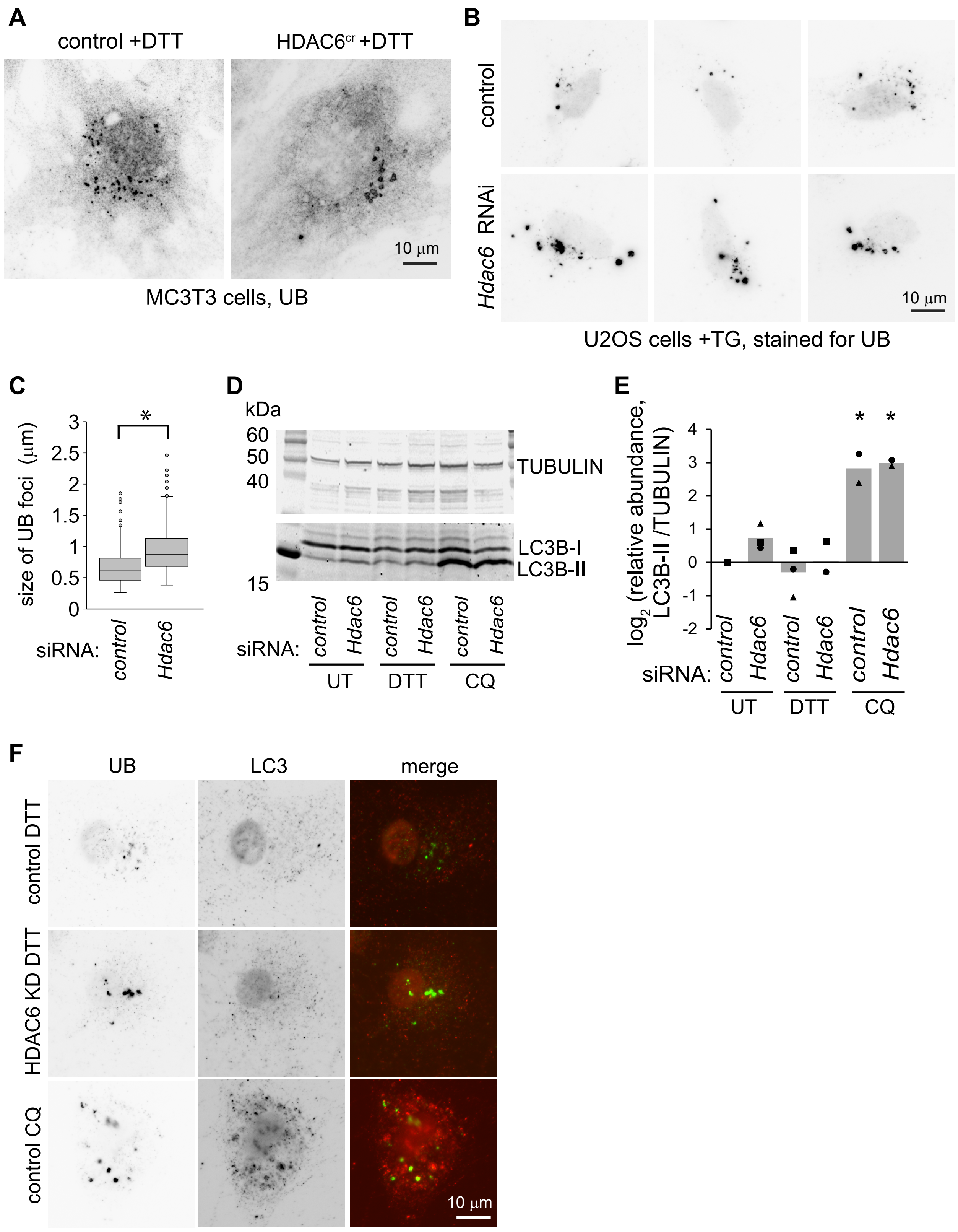

Piscopo et al, Fig S1

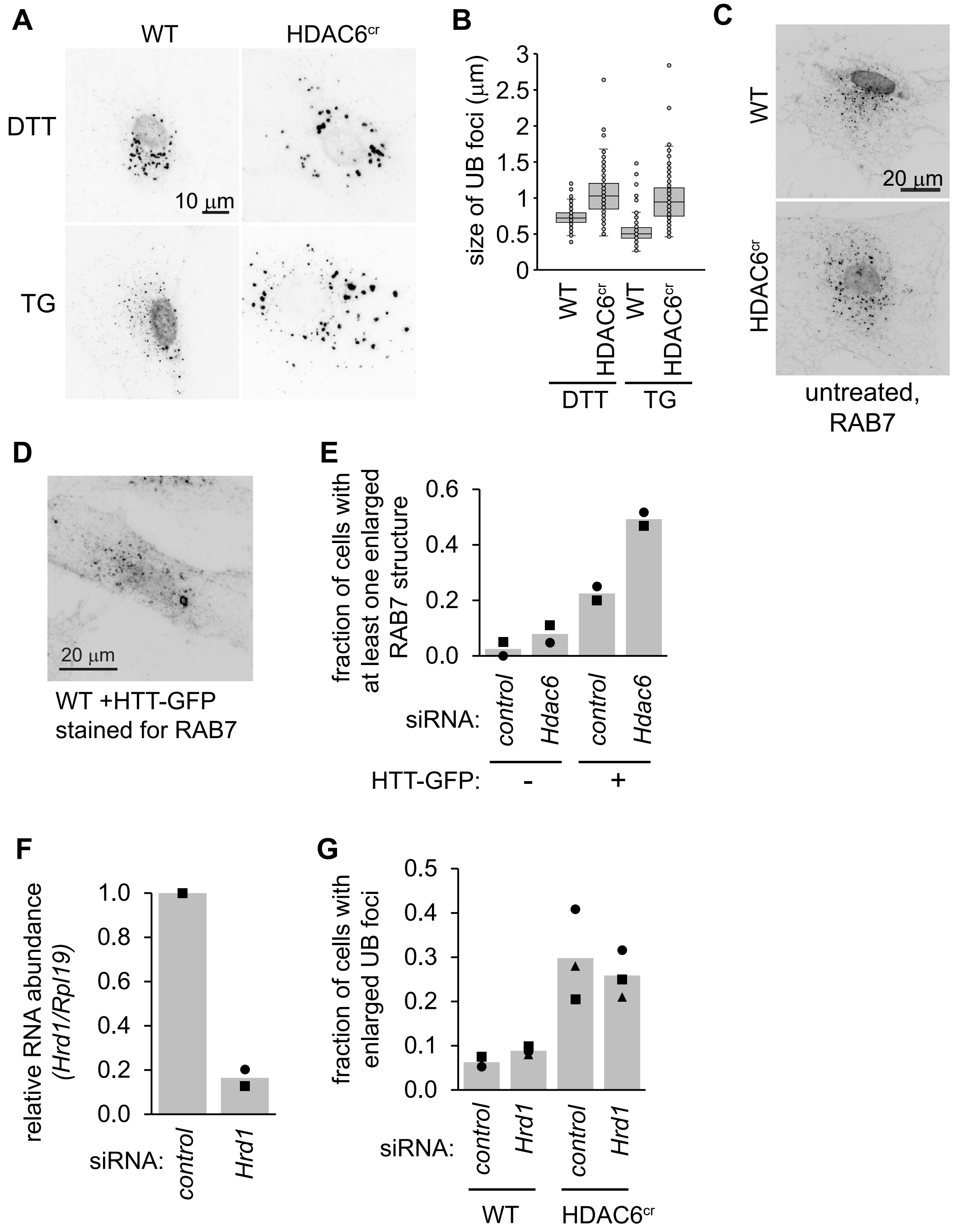
A
WT
HDAC6cr
DTT
TG
10 µm
B
size of UB foci (µm)
3
2.5
2
1.5
1
0.5
0
WT
HDAC6cr
WT
HDAC6cr
DTT
TG
C
WT
20 µm
HDAC6cr
untreated, RAB7
D
20 µm
WT +HTT-GFP stained for RAB7
E
fraction of cells with at least one enlarged RAB7 structure
0.6
0.4
0.2
0.0
siRNA:
control
Hdac6
control
Hdac6
HTT-GFP:
-
+
F
relative RNA abundance (Hrd1/Rpl19)
1.0
0.8
0.6
0.4
0.2
0.0
siRNA:
control
Hrd1
G
fraction of cells with enlarged UB foci
0.5
0.4
0.3
0.2
0.1
0.0
siRNA:
control
Hrd1
control
Hrd1
WT
HDAC6cr

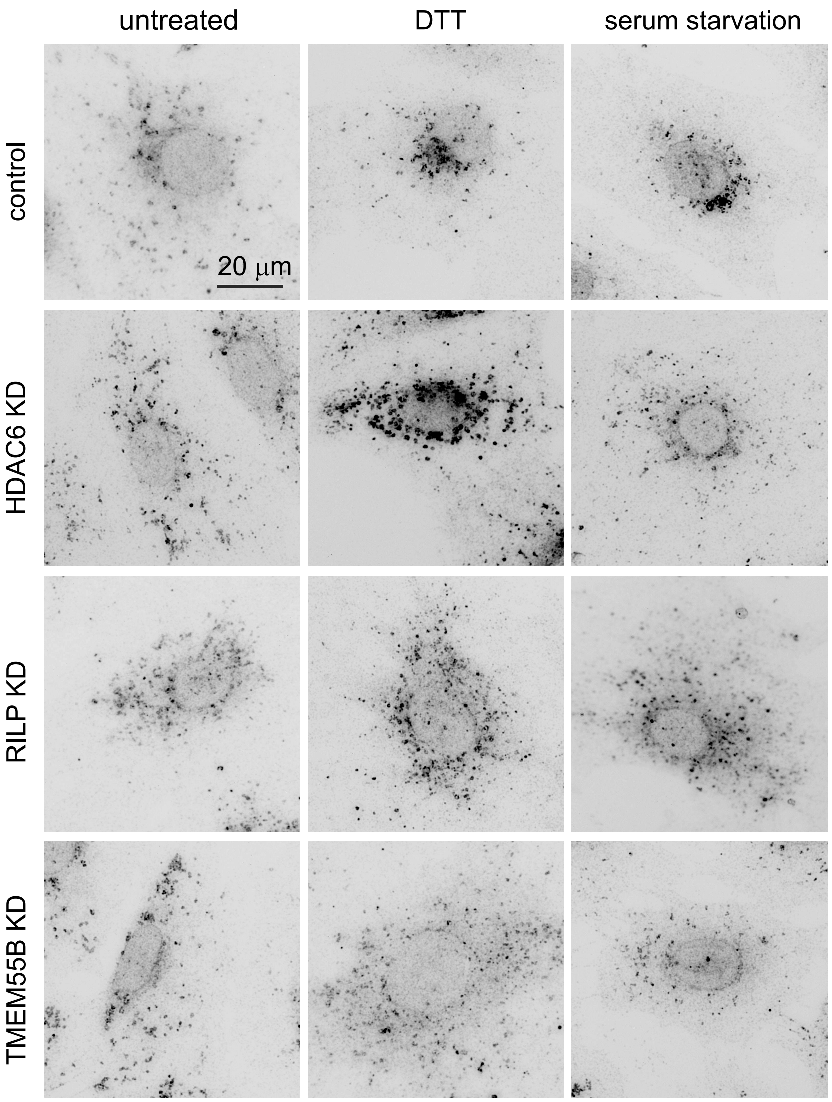

Piscopo et al, Fig S3
